# Different Responses in Root Water Uptake of Summer Maize to Planting Density and Nitrogen Fertilization

**DOI:** 10.3389/fpls.2022.918043

**Published:** 2022-06-24

**Authors:** Yang Gao, Jinsai Chen, Guangshuai Wang, Zhandong Liu, Weihao Sun, Yingying Zhang, Xiaoxian Zhang

**Affiliations:** ^1^Farmland Irrigation Research Institute, Chinese Academy of Agricultural Sciences, Xinxiang, China; ^2^Department Sustainable Agriculture Science, Rothamsted Research, Harpenden, United Kingdom

**Keywords:** stable isotopes, summer maize, Bayesian inference method, planting density, fertilization

## Abstract

Modifying farming practices combined with breeding has the potential to improve water and nutrient use efficiency by regulating root growth, but achieving this goal requires phenotyping the roots, including their architecture and ability to take up water and nutrients from different soil layers. This is challenging due to the difficulty of *in situ* root measurement and opaqueness of the soil. Using stable isotopes and soil coring, we calculated the change in root water uptake of summer maize in response to planting density and nitrogen fertilization in a 2-year field experiment. We periodically measured root-length density, soil moisture content, and stable isotopes δ^18^O and δD in the plant stem, soil water, and precipitation concurrently and calculated the root water uptake based on the mass balance of the isotopes and the Bayesian inference method coupled with the Markov Chain Monte Carlo simulation. The results show that the root water uptake increased asymptotically with root-length density and that nitrogen application affected the locations in soil from which the roots acquired water more significantly than planting density. In particular, we find that reducing nitrogen application promoted root penetration to access subsoil nutrients and consequently enhanced their water uptake from the subsoil, while increasing planting density benefited water uptake of the roots in the topsoil. These findings reveal that it is possible to manipulate plant density and fertilization to improve water and nutrient use efficiency of the summer maize and the results thus have imperative implications for agricultural production.

## Introduction

Maize (*Zea mays* L.) is a staple crop that is economically significant and most produced ahead of wheat (Hodgkinson et al., 2017). Since the available croplands and water resources for agriculture have both been dwindling, maize production is facing an unprecedented challenge due to its high demand for water and nutrients. Improving soil resource use efficiency is hence critical to sustaining maize production to feed an increasing global population projected to reach 9 billion in 2050 (Giri et al., 2018).

Crop management has substantial effects on spatiotemporal availability of soil resources, as well as root growth and its acquisition of water and nutrients (Chapman et al., 2012; Peake et al., 2013; Guan et al., 2014). Modifying farming practices to coordinate roots to grow in a way that facilitates water and nutrient acquisition is thus attractive and has been studied intensively over the past two decades (Widdicombe and Thelen, 2002; Liu et al., 2010, 2021; Ramezani et al., 2011; Li et al., 2015; Prechsl et al., 2015; Wu et al., 2015; Piao et al., 2016; Wang et al., 2017b). Although the experimental results are not conclusive, a consensus is that cultivars with “deep, steep, and cheap” root traits are effective for accessing resources in the subsoil thereby achieving high yield under terminal or intermittent drought conditions, whereas under conditions without water and nitrogen stresses, enhancing proliferation of thin lateral roots in the topsoil benefits resource acquisition and yield (Lynch, 2013). This is corroborated by experiments over the past decade. For example, Wang et al. (2019) found that reducing nitrogen application proliferated fine roots of maize in the subsoil, thereby enhancing its water use efficiency when the crop was under moderate water stress, and Ma and Song (2016) reported that adjusting fertilizer application reshaped root-length distribution of maize and impacted its water uptake from the soil profile as a result.

Along with fertilization, planting density and tillage also alter root growth and change the way crops take up water and nutrients from soil (Li et al., 2017b; Fiorini et al., 2018; Shao et al., 2018; Zhang et al., 2018). The impact of farming practice on soil resource acquisition has been well-documented (Majdi and Andersson, 2005; Kou et al., 2017; Li et al., 2017a), but the role played by root adaptation and the associated uptake of water and nutrient is not well-established due to the difficulty of *in situ* root phenotyping in opaque soils (Whalley et al., 2017). Traditional root measurement involving soil excavation is not only labor-intensive and time-consuming, but it is also insufficient to quantify water uptake as root-length density is not necessarily proportional to soil resource acquisition (Meinzer et al., 2001). Measuring soil-dying is another method to quantify root uptake (Whalley et al., 2017), but it is reliable only when soil is relatively dry where water dynamics is predominantly induced by root water uptake. It is inadequate when soil is wet following irrigation or rainfall in which soil-water redistribution becomes significant.

The impact of planting density and fertilization on crop yield has been fairly studied, but there is a lack of understanding of their impact on root growth and the consequence for water and nutrient uptake (Testa et al., 2016; Li et al., 2021; Liu et al., 2021). For example, it remains elusive that to what extent a change in fertilization and planting pattern alters root growth and water and nutrient uptake from different soil layers (Goebel et al., 2015; Du et al., 2018; Penna et al., 2020). Irrigation is known to influence root water uptake patterns significantly (Yang et al., 2015a; Ma and Song, 2016; Wu et al., 2016, 2018b; Zheng et al., 2019), while in semi-arid regions, such as northern China, maize is often grown rain-fed. Improving planting density and fertilization and understanding their combined impact on root growth and water acquisition is paramount to safeguard maize production in these regions but has been overlooked. The purpose of this article is to bridge this knowledge gap. A 2-year field experiment has been conducted with different combinations of nitrogen (N) fertilization and plant patterns. During the experiment, we periodically measured soil moisture content, root-length density, as well as the concentration of δ^18^O and δD in soil water, plant stem, and precipitation, concurrently. Root water uptake from different soil layers was calculated based on the intersection method and mass balance of the isotopes coupled with the Bayesian inference method (Eggemeyer et al., 2009; Brooks et al., 2010; Zhang et al., 2011; West et al., 2012; Schwendenmann et al., 2015; Yang et al., 2015b; Ma and Song, 2016). We analyzed how N fertilization and planting patterns combined to modulate root water uptake from different soil layers, as well as the underlying mechanisms.

## Materials and Methods

### Experimental Site and Planting

The 2-year field experiment was conducted in 2016 and 2017 at the Experimental Station of Institute of Farmland Irrigation, Chinese Academy of Agricultural Science (CAAS), located at Qiliying (35^0^08′ N, 113^0^45′ E and 81 m altitude) in Xinxiang, Henan province, China. The mean annual rainfall and temperature (1951–2014) at the station are 578 mm and 14.3°C, respectively, and the average precipitation over the growing season of the maize (June to September) is 418 mm. The physical and chemical properties of the soil are given in Table 1. During the experiment, the groundwater table was >5 m below the ground surface and had negligible impacts on the crop.

**Table 1:** Physical and hydraulic parameters of the soil at the experimental station.

**Soil depth (cm)**	**Particle size distribution (%)**			**Texture**	**B.D. (g cm^**−3**^)**	***θ_*FC*_* (cm^3^ cm^−3^)**	***θ_*s*_* (cm^3^ cm^−3^)**	***K_***s***_* (cm day^**−1**^)**
	**Clay**	**Silt**	**Sand**					
0–20	3.80	43.14	53.06	Sandy loam	1.56	0.341	0.409	55.85
40–60	6.06	48.33	45.61	Sandy loam	1.54	0.327	0.409	43.98
60–80	4.55	47.49	47.96	Sandy loam	1.42	0.283	0.412	52.57
80–100	1.57	16.95	81.48	Loamy sand	1.45	0.294	0.393	131.20
Average	4.52	40.27	55.21	Sandy loam	1.51	0.310	0.403	51.81

*B.D, bulk density; θ_FC_, field capacity; θ_s_, saturated water content; K_s_, saturated hydraulic conductivity*.

**Figure 1:**
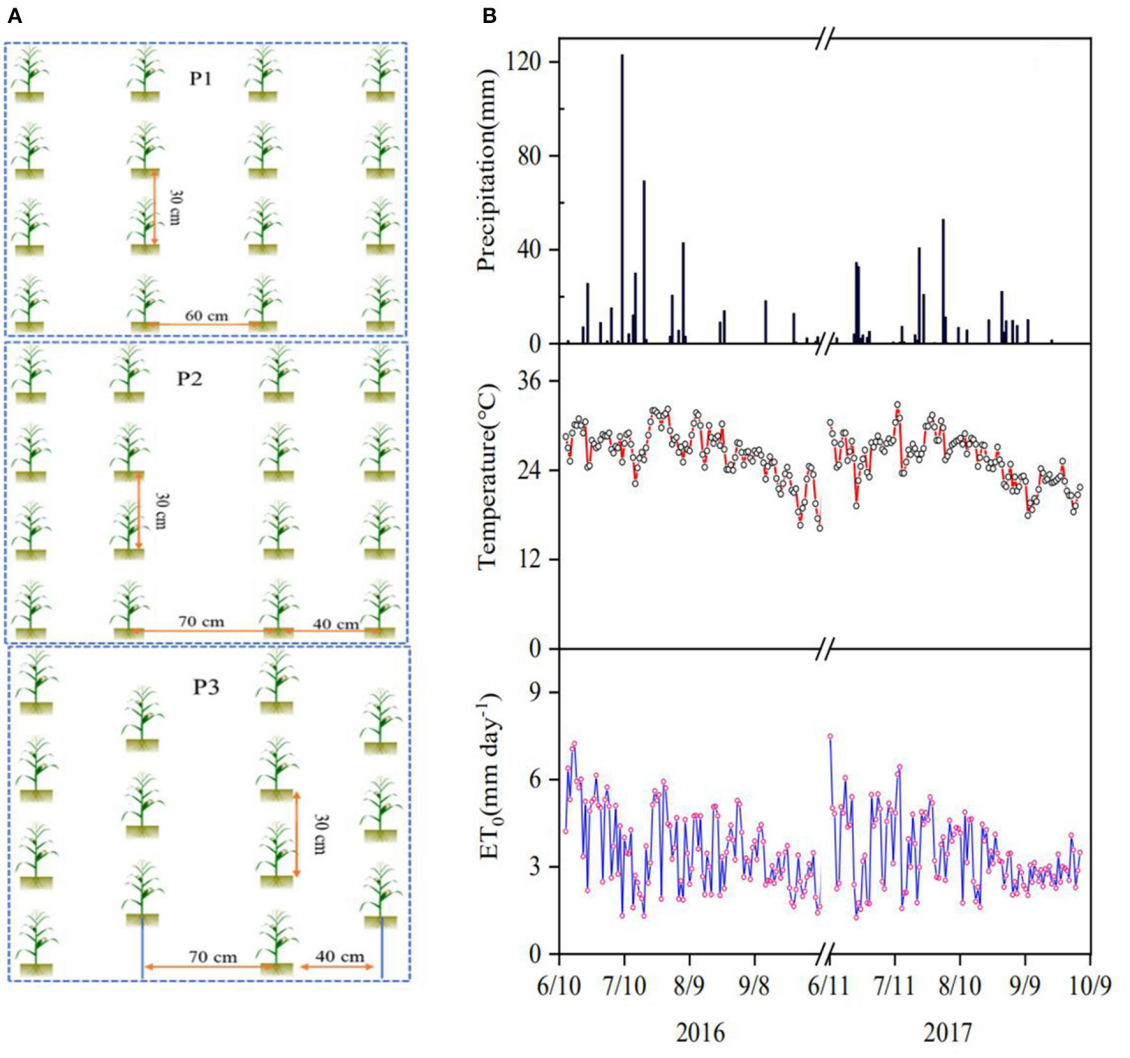
**(A)** Sketch of the three planting patterns: P1 - Constant 60 cm inter-row spacing; P2 – alternate 40 cm and 70 cm inter-row spacing, and P3 - zig-zag pattern with alternate 40 cm and 70 cm inter-row spacing; the intra-row spacing is 30 cm in all treatments. **(B)** Changes in air temperature, reference evapotranspiration (*ET*_0_) and precipitation in the 2-year experimental period.

The maize variety Denghai-605 (*Zea mays* L.) was used as the model plant and the experiment consisted of three planting patterns and two nitrogen applications, each having three replicates, on a number of 6 m× 18 m plots designed using the randomized complete block method. The seeds were sown on June 13, 2016, and June 10, 2017, and the associated harvest was on October 8, 2016, and October 4, 2017, respectively. The intra-row planting distance was 30 cm in all treatments, and the treatments differed in the inter-row spacing and pattern as shown in Figure 1A: constant 60 cm spacing (P1); alternate 40 cm and 70 cm spacing (P2); zig-zag pattern with alternate 40 cm and 70 cm spacing (P3). The planting densities associated with P1, P2, and P3 were 5.6 × 10^4^, 6.1 × 10^4^, and 7.3 × 10^4^ plants ha^−1^, respectively. Each planting pattern had two N applications: 240 kg N ha^−1^ (N_240_) and 120 kg N ha^−1^ (N_120_) to cover the varying N applications used by the local farmers (Zhang et al., 2021); in each treatment, 50% of N fertilizer was broadcasted with 90 kg ha^−1^ of P_2_O_5_ and 75 kg ha^−1^ of K_2_O as basal fertilizer over each plot, followed by immediate plowing to avoid nitrogen volatilization; the remaining 50% of N fertilizer was applied as top-dressing at early jointing stage in the middle of July. All plots were irrigated with 60 mm of water on June 18 in 2016 and June 15 in 2017, using sprinkler irrigation to facilitate seed germination and emergence.

### Sampling

Plant and soil in each plot were concurrently sampled on June 24, July 14, August 3 and 22, September 12 and 30 in 2016, and on June 23, July 13 and 30, August 22, and September 10 and 27 in 2017. During each sampling day, three plants in each plot were randomly sampled first and the stem epidermis of each sampled plant was then immediately removed to avoid potential contamination by isotopically enriched water (Querejeta et al., 2007). The stem was cut into 4–5 cm segments before being frozen in a sealed vial for isotope analysis. Following the plant sampling, soil samples at the depths of 5, 10, 20, 40, 60, 80, and 100 cm were taken from three sites close (about 5 cm) to each sampled plant using a hand-driven auger. Soils taken from the same depths from the three sites were pooled, a half of the pooled soils was used for isotope analysis, and the remaining was used for soil moisture measurement using the gravimetric method.

The soil and plant samples for stable isotope analysis were kept in a freezer at −20°C. Prior to the analysis, water in the stem and the soil water was extracted using a vacuum extraction system (LI-2000, LICA, China) by applying a suction pressure for 0.5–1.5 h. The results revealed this had extracted more than 99.0% of the water in both soil and stem and was therefore deemed adequate to obtain the unfractionated water from the soil and stem for isotope analysis (West et al., 2006; Meißner et al., 2014).

The following day after the soil and plant sampling, further soil cores were taken down to 100 cm deep at 10-cm intervals close to each sampled plant using an auger with an internal diameter of 6.91 cm and an external diameter of 7 cm to measure root-length density. Each cored sample was transferred to a sieve (0.5 mm mesh), and the sieve was then suspended in a water-filled trough to wash the soil away at low pressure to leave the root segments only. The root-length density (RLD) in each sample was analyzed using the WinRHIZO Reg. 2007d (Regent Instrument Inc.) based on the images of the roots scanned using the EPSON PREFECTION_TM_ V700 Photo Flatbed Scanner at resolution of 6,400 dpi × 9,600 dpi. The RLD was calculated as the ratio of the lengths of all root segments in each core to the volume of the core (375.0 cm^3^). The root-length densities for samples taken from each soil profile were normalized by the total root lengths in all samples taken from the soil profile.

Precipitation was sampled from a funnel filled with a ping-pong ball inside to prevent evaporation. A polyethylene bottle was connected to the bottom of the funnel to collect raindrops. It was difficult to collect sufficient rainwater, as well as strong fractionation, when precipitation was <5 mm, and we hence only considered precipitation events which were >5 mm. The irrigation water was sampled for helping data interpretation. To prevent isotope fractionation caused by evaporation, both rain and irrigation water samples were stored in an air-tight container at 4°C prior to isotope analysis.

### Isotope Analysis

All water samples were analyzed for deuterium (δ^2^H) and oxygen (δ^18^O). We compared the isotopes in the water samples by injecting the water into a high-temperature conversion/elemental analyzer (TC/EA) coupled with a Con-Flo III interface on a Delta V Advantage isotope ratio mass spectrometer (Thermo-Electron Corporation, Bremen, Germany). All water samples were calibrated and normalized to an internal laboratory water standard previously calibrated against the Vienna Standard Mean Ocean water (VSMOW, 0‰). The results were expressed as δ-value related to the VSMOW:


(1) $$\delta (\%0)\;=\;\left(\frac{R_{sample}}{R_{VSMOW}}\;-\;1\right)1000$$

where *R* is the ^18^O/^16^O or ^2^H/^1^H ratio with the subscript “sample” and “VSMOW” referring to the value of the samples and of the Vienna Standard Mean Ocean water, respectively. The error of the measurements was ±1.4‰ for δ^2^H and ±0.1‰ for δ^18^O.

### Evapotranspiration

Daily precipitation, average temperature, relative humidity, sunshine, and wind speed were recorded from an automatic weather station installed on the site (RX3000, HOBO, USA). The reference evapotranspiration, *ET*_*o*_ (mm d^−1^), was calculated from the Penman–Monteith equation (Allen et al., 1998):


(2) $$\begin{array}{r} ET_{o}\;=\;\frac{0.408\Delta \left(R_{n}-G\right)\gamma \frac{900}{T\;+\;273}u_{2}\left(e_{s}-e_{a}\right)}{\Delta +\gamma \left(1\;+\;0.34u_{2}\right)} \end{array}$$

where *R*_*n*_ is the net radiation (MJ m^−2^ d^−1^), *G* is the soil heat flux density (MJ m^−2^ d^−1^), *T* (^o^C) and *u*_2_ (m s^−1^) are the mean daily air temperature and wind speed at 2 m above the ground surface, respectively, *e*_*s*_ and *e*_*a*_ are the saturated and actual vapor pressure (kPa), respectively, Δ is the slope of saturation vapor pressure curve (kPa ^o^C^−1^), and γ is the psychrometric constant (kPa ^o^C^−1^).

### Root Water Uptake

There is no isotopic fraction when water flows from soil into roots (Dawson and Ehleringer, 1991; Ehleringer and Dawson, 1992), and we hence use mass balance to track the origin of the isotopes in the plant stem. We divided the soil profile into N layers, assuming all water taken up by the roots came from these layers. During a time period of Δ*t*, if the transpiration is *S* and the water taken up by the roots from i_th_ soil layer is*S*_*i*_, from the mass balance we have


(3) $$\begin{array}{l} \begin{array}{c} \begin{array}{l} \delta D_{stem}={\sum^{\text{​}}}_{i=1}^{N}f_{i}\cdot \delta D_{i},\\ \delta^{18}O_{stem}={\sum^{\text{​}}}_{i=1}^{N}f_{i}\cdot \delta^{18}O_{i}, \end{array} \end{array}\\ {\sum^{\text{​}}}_{i=1}^{N}f_{i}=1,\\ f_{i}=S_{i}/S, \end{array}$$

where δ*D*_*stem*_and $\delta^{18}O_{stem}$are the concentration of H^2^ and O^18^ measured from the stem water, respectively, δ*D*_*i*_and $\delta^{18}O_{i}$ are their counterparts in the ith soil layer, and *f*_*i*_ is the fraction of the water taken up by roots in the ith soil layer. If the two isotopes are not correlated and the soil profile is divided into three layers, Equation (3) can be solved exactly. However, given the irregular distribution of isotopes over the soil profile, to be consistent with the root density measurement, we divided the soil profile into four uneven layers: 0–5 cm, 5–20 cm, 20–60 cm, and 80–100 cm, calculating the water uptake of roots in each layer. There are more variables than the number of equations, and we hence solved Equation (3) using the Bayesian inference method, which gives the most likelihood rather than the exact amount of water taken up by roots in each layer.

Given a set of measurements ***Y***, the Bayesian formalism postulates the problem as follows:


(4) $$\begin{array}{r} p\left(\text{f}|\text{Y}\right)=\frac{p\left(\text{f}\right)p\left(\text{Y}|\text{f}\right)}{p\left(\text{Y}\right)}\propto p\left(\text{f}\right)L\left(\text{f}|\text{Y}\right), \end{array}$$

where*p*(f|Y)and*p*(f)represent the posterior and prior distributions of the normalized root uptake from each soil layer, *p*(Y|f)and*p*(*Y*)are the posterior and priori distributions of the measured isotopes, and*L*(f|Y) is the likelihood function, respectively; *L*(f|Y) was estimated using the measured data as follows assuming that the measured isotopes were normally distributed


(5) $$\begin{array}{r} L\left(\text{f}|\text{Y}\right)=\overset{N}{\underset{t=1}{\prod }}\frac{1}{\sqrt{2\pi \sigma_{t}^{2}}}\exp\left[\frac{1}{2\sigma_{t}^{2}}{\left(\frac{y_{t}-y_{t}\left(\text{f}\right)}{\sigma_{t}^{}}\right)}^{2}\right], \end{array}$$

where*y*_*t*_and*y*_*t*_(f)are the measured and calculated isotopes in the plant stem using Equation (4), respectively, and$\sigma_{t}^{2}$ is the variance. The posterior distribution of the root uptake from all soil layers defined in Equation (5) was calculated numerically based on the Monte Carlo Markov chain simulation, using the open-source SIAR software [CRAN—Package siar (r-project.org)] (Parnell et al., 2013).

### Statistical Analysis

The Kolmogorov–Smirnov (K-S) test showed that the isotopes measured for the plant stem and soil water were normally distributed and these distributions were used to calculate the above likelihood function. We used hierarchical cluster analysis to classify the soil water and the analysis of variance (ANOVA) to investigate the difference in root water uptake, δ^18^O and δD in water, as well as the difference between treatments with *p* < 0.05 deemed significance. Multiple comparisons were made using the least significant difference (LSD) to quantify significant variation in δ^18^O and δD in the soil profile. All statistical analyses were performed using the SPSS 21.0 program.

## Results

### Environmental Factors and Isotopes in Precipitation

**Figure 2:**
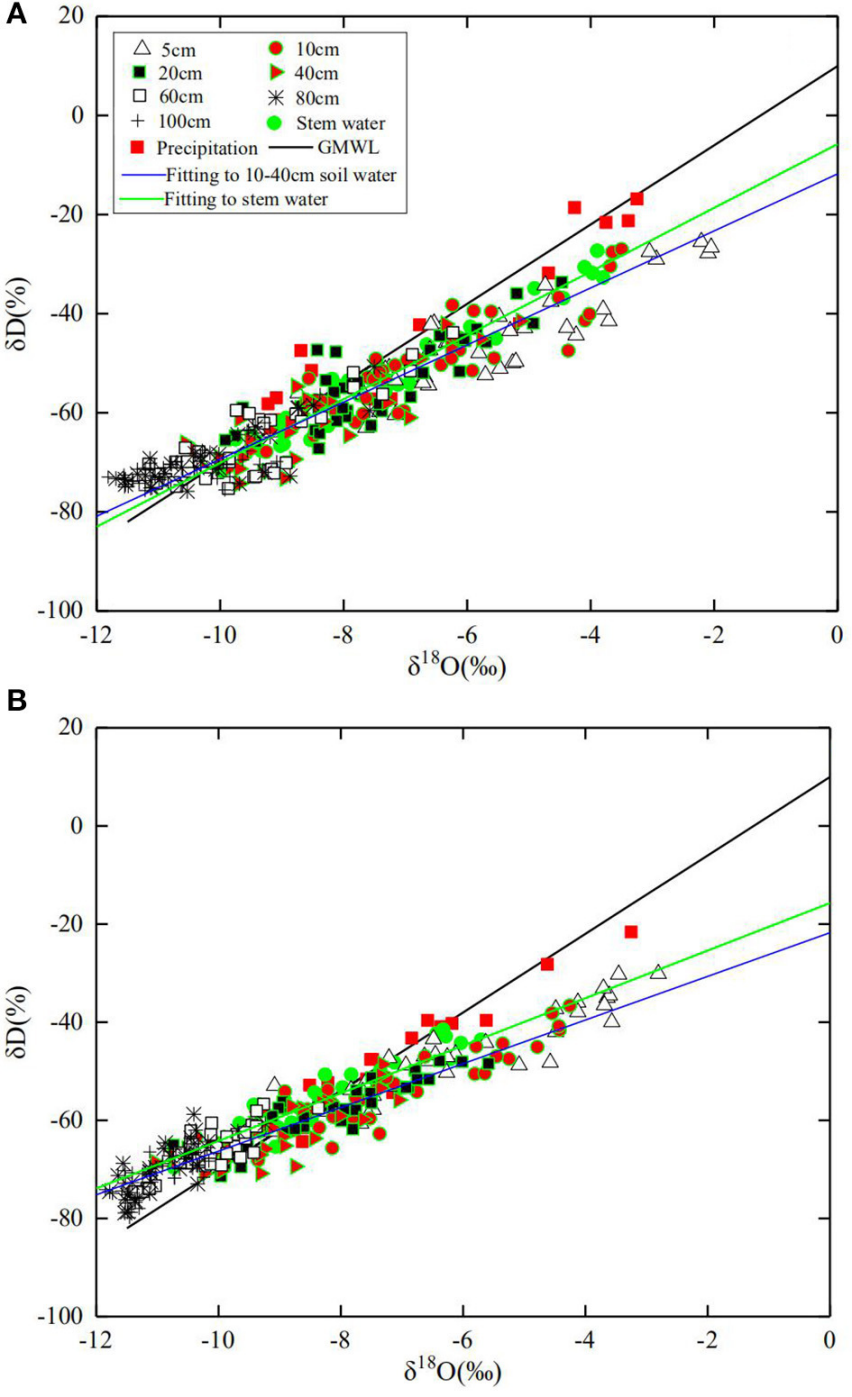
The relationship between δ^18^O and δD for data measured from all water samples in comparison with the global meteoric water line (GMWL) in 2016 **(A)**, and 2017 **(B)**, as well as the linear fittings to stem water and water in the 10–40 cm of soil in all treatments respectively.

The changes in precipitation, air temperature, and *ET*_*o*_ during the experimental period are given in Figure 1B. The daily average *ET*_*o*_ and temperature were 3.6 mm d^−1^ and 26.4°C in 2016 and 3.5 mm d^−1^ and 25.4°C in 2017, respectively. The seasonal rainfall in 2016 and 2017 was 438.9 mm and 319.6 mm, respectively, during the experimental period; there were 15 and 17 precipitation events with rainfall >5 mm in 2016 and 2017, accounting for 95% and 92% of the precipitation in each year, respectively. During the experiment, the δD measured from the precipitation ranged from −64.34 to −16.83% with a mean of −45.37%, and the δ^18^O varied from −9.39% to −3.25% with a mean of −6.92% (Figure 2). The local meteoric water line (*LMWL*) measured from the precipitations was approximately linear: δ*D* = 6.7935δ^18^*O*+2.0593 (R^2^ = 0.88) for 2016 and δ*D* = 6.7818δ^18^*O*+1.2127 (R^2^ = 0.91) for 2017.

### Isotopes in Soil Water and Stem Water

The isotopes measured from the stem water varied with time significantly in all treatments (Figure 2 and Supplementary Figures 1, 2), implying an impact on the environment. The relationship between δ^18^O and δD was fitted to δ*D* = 6.4326δ^18^*O*-5.7838 (R^2^ = 0.95) and δ*D* = 5.0586δ^18^*O*-17.289 (R^2^ = 0.89) for 2016 and 2017, respectively. Precipitation and irrigation altered isotopes in soil water, with the measured δ^18^O declining along the soil depth (Supplementary Figures 1, 2). The δ^18^O and δD in soil water fall into two groups, each following its own linear relationship (Figure 2). Samples taken from the 10–40-cm soil layer with δ^18^O>-10% were consistent with the δ^18^O -δD relationship for the stem water, suggesting that most water taken up by the roots originated from this soil layer. In contrast, data with δ^18^O <-10% for samples taken from 60- to 100-cm soil layer deviated from the δ^18^O -δD relationship for the stem water, indicating that they are unlikely the main source of the water respired by the crop. As a comparison, the linear fitting curve for the stem water and the water in the 10–40-cm soil layer is also plotted in Figure 2.

### Root-Length Density and Root Water Uptake

**Figure 3:**
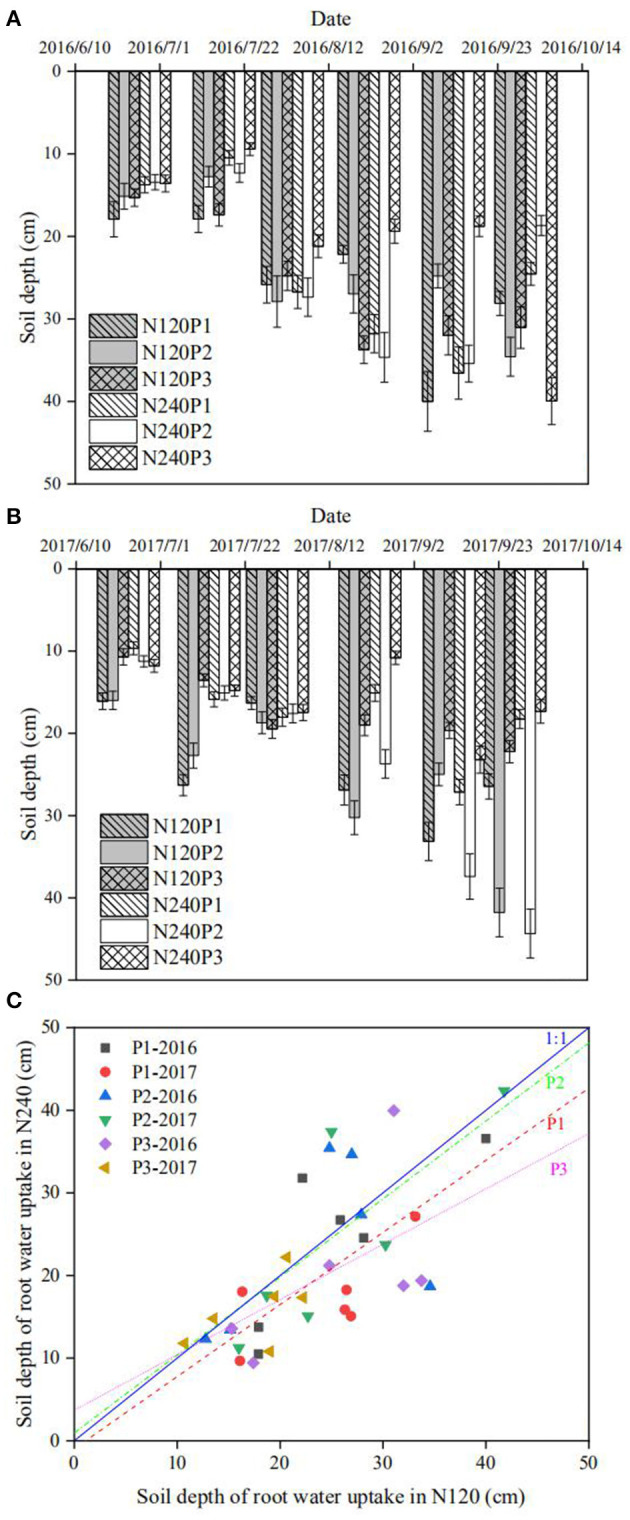
Spatiotemporal changes in root uptake depth estimated from the intersection method in 2016 **(A)**, and 2017 **(B)**. The impact of different combinations of N fertilization and planting density on root water uptake depth estimated using the intersection method for all treatments over the 2-year experiment **(C)**.

The region in soil where the roots took up water can be estimated by plotting δ^18^O (or δD) measured from the stem water along the soil profile. Its intersections with the distribution of δ^18^O (or δD) in the soil water are the points around which the roots took up the water (Figures 3A,B, Supplementary Figures 1, 2). While uncertainties might be raised when there are more than one such intersection, these did not appear in our experiment. The roots took most of the water from soil around the depth of 10 cm at the seedling stage, and they then progressively moved downward to the depth of 40 cm during the flowering and harvesting stages (Figures 3A,B, and Supplementary Figures 1, 2).

**Figure 4:**
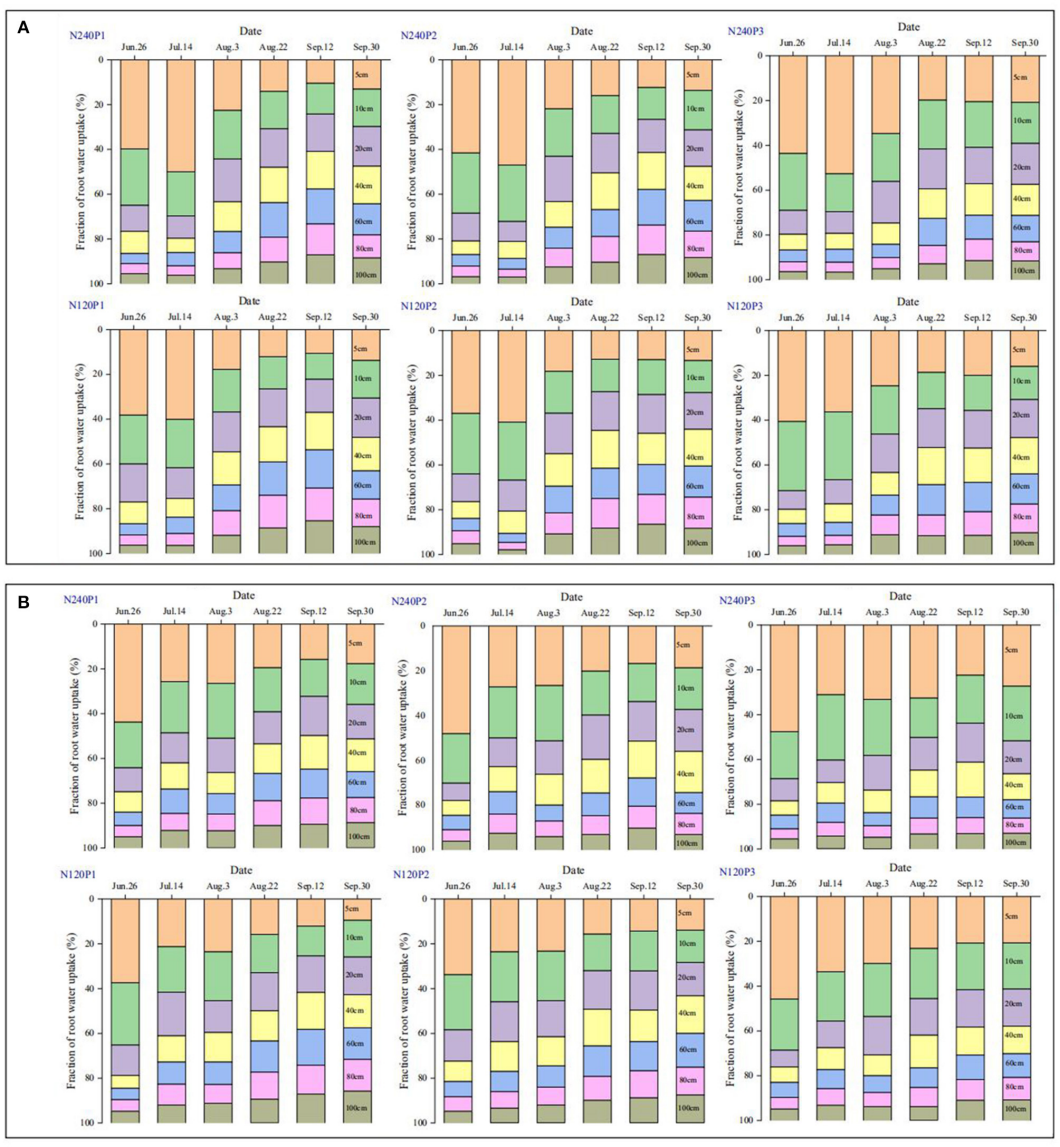
Spatiotemporal changes in the normalized root water uptake rate under different treatments (see Figure 1A) calculated based on the mass balance of isotopes and the Bayesian inference method; 2016 **(A)**, and 2017 **(B)**.

The intersection method illustrates the approximate regions from which the roots take up water. To quantify the contribution of each soil layer to the transpiration of the crop at different growing stages, we estimated the posterior distribution of the root water uptake from each layer. The posterior distribution for each soil layer is a probability distribution of the root water uptake, and we present the mean only as this is the most likely water that the roots in this layer had taken up (Figure 4).

In the early stage before the N top-dressing (13 July in 2016 and 15 July in 2017), about 60% of the transpired water emanated from the top 10 cm of soil, while following the top-dressing, the water uptake from the subsoil increased steadily. The timing in 2016 at which the water uptake from the subsoil (20–60-cm layer) exceeded that from the topsoil was in the late August for N240, while for N120 this was delayed to the early August (Figure 4A). For P1 and P2 in 2017, the difference in root water uptake between the topsoil and the subsoil was similar to that in 2016. For P3, root water uptake from the topsoil was higher than that from the subsoil during the whole growing period (Figure 4B); even at the late stage, the roots in the top 10 cm of soil contributed more than 30% of the total transpiration. The relative significance of water uptake from different soil layers varied between the 2 years, but the trend was the same: N top-dressing increased the water uptake from the subsoil, and when the planting density (P1 and P2) and N fertilization were both low, the water uptake rate of roots in the 20–60-cm soil layer was higher than that in the top 10-cm soil layer.

**Figure 5:**
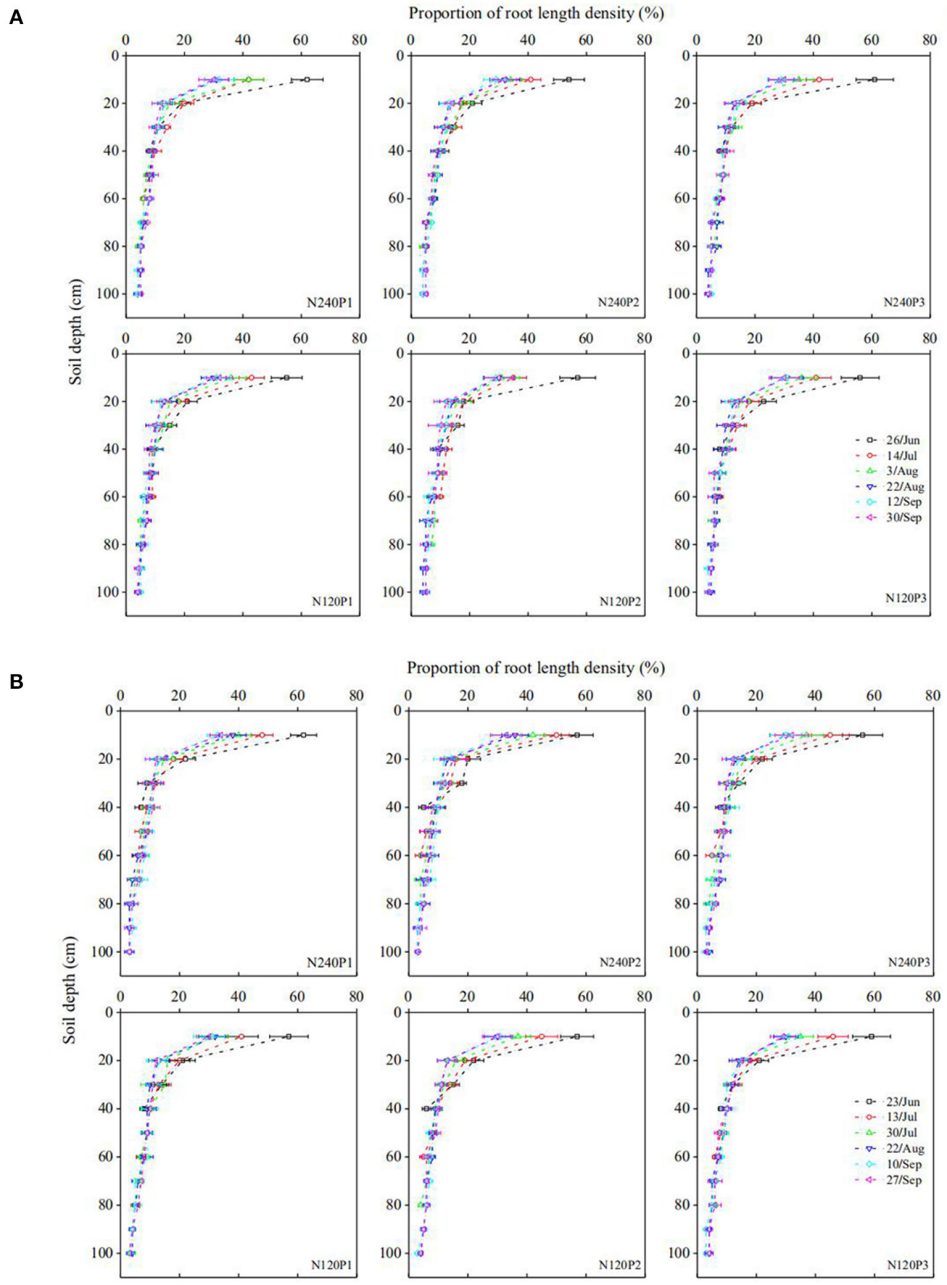
Spatiotemporal variation in the normalized root density under different treatments (see Figure 1A) in 2016 **(A)**, and 2017 **(B)**.

**Figure 6:**
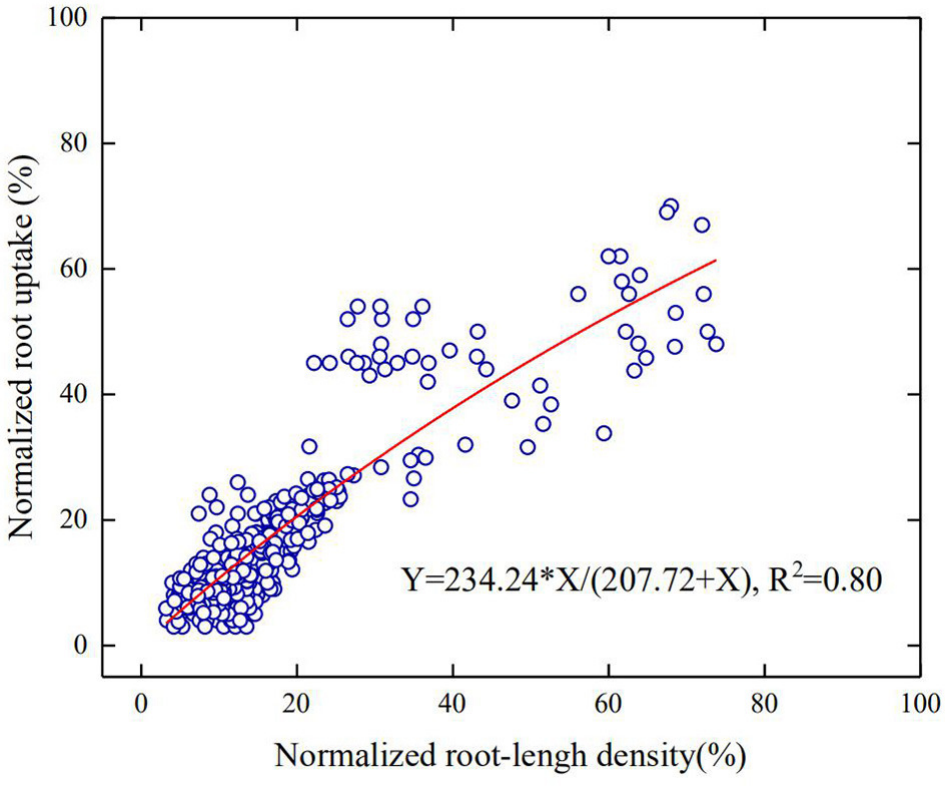
The increase in the normalized root water uptake rate with the normalized root-length density for all treatments in the 2-year experiment.

Water uptake of a root segment is proportional to the difference between water potential on the soil-root surface and in its xylem network. For a region where roots are sparse, the roots are unlikely to interfere with each other and water uptake from this region is hence proportional to root-length density. In contrast, for regions where the roots are dense, continuous water uptake drives distant water moving into the rhizosphere; when the influencing zone of the roots meets, the roots start competition for water. Therefore, in general, root water uptake in a soil profile increases with the root-length density asymptotically rather than linearly. At different growth stages, the root-length density in all treatments decreases approximately exponentially with the soil depth (Figure 5). Pooling the root water uptake calculated using the statistical method and the measured root-length density at different growing stages in all treatments, Figure 6 shows that the root water uptake rate (*y*) calculated using the statistical methods increased asymptotically with the normalized root-length density (*S*). The increase is fitted to*y* = *kS*/(*A*+*S*), with *k* = 234.24 and *A* = 207.72. The slight deviation from linear increase when root-length density is high (in the topsoil) indicates the existence of competition between roots in the topsoil for water (Figure 5).

### N Fertilization and Planting Pattern Effects

To elucidate the combined impact of fertilization and planting density on root water uptake, we plotted in Figure 3C the depths estimated from the cross-points between the isotopes in soil profile and the stem water as shown in Supplementary Figures 1, 2 (called root water uptake depth hereafter) under different treatments. It is manifest that, except for a few points measured following the N top-dressing, the majority of the depths are below the 1:1 line, indicating that reducing N fertilization from 240 to 120 kg N ha^−1^ increased water uptake from the subsoil. Linearly fitting the data for the same planting pattern reveals that the fitting for P3 (most densely planted) deviates from the 1:1 line more significantly, followed by P2 though the difference between P1 and P2 is not significant. These phenomena suggest that the impact of N fertilization and planting density on root water uptake is confounded. This is corroborated by the results calculated from the statistical method (Figure 4).

The impact of basal fertilization and planting density on root water uptake from different soil layers appeared to be minor in the early stage, and significant differences started to emerge after the N top-dressing (Figure 4). Reducing N application promoted root penetration to take more water from the subsoil (Figure 4). For example, for P1 in the middle of September 2016, the roots in the 80–100-cm soil layer contributed 30% of the respired water under N_120_, while under N_240_ the same soil layer contributed 25% of the transpired water (Figure 4A). For P3 (the highest planting density) combined with N_120_, the critical depth below which the roots contributed <10% of the transpired water increased from 40 cm in the early August to around 100 cm at the end of September. In contrast, during the same period but under the combination of P3 and N_240_, the critical depth below which the roots contributed <10% of the transpired water increased from 40 to 60 cm only (Figure 4A). The results in 2017 did not replicate those in 2016, but the trend of the response of root water uptake to planting pattern and N fertilization was the same (Figure 4B).

## Discussion

### Changes in Isotopes

The slope of the *LMWL* was smaller than that of the global meteoric water line δ*D* = 8δ^18^*O*+10 (Craig, 1961), indicating that humidity change and secondary evaporation might have enriched the O^18^ in the precipitation in our experimental site (Araguás-Araguás et al., 1998). Most δ^18^O and δD in soil water were plotted beneath the LMWL (Figure 2), implying that soil water with its origin from the rainfall was likely to have undergone evaporation which enriched δ^18^O (Wang et al., 2017a). The significant difference was found in isotopes in soil water between the treatments (Supplementary Figures 1, 2) due to the effects of rainfall and root water uptake. The heavy rain (123 mm) on July 9, 2016, (Figure 1B) enriched the isotopes, with δ^18^O measured from soil water varied approximately 1-fold along the soil profile (Supplementary Figure 1). Soil evaporation enriched δ^18^O in the vicinity of the soil surface, and δ^18^O in the topsoil fluctuated seasonally due to the periodic precipitation (Tang and Feng, 2001; McCole and Stern, 2007; Dai et al., 2015; Sprenger et al., 2016; Wu et al., 2018a). Our results agreed with those found in Tang and Feng (2001) and Wu et al. (2018a), all showing that hydrological processes modulated the seasonal change of isotopes in the topsoil water.

The δD and δ^18^O in the precipitation and stem water were linearly correlated, but this correlation does not apply to soil water (Figure 2). The δD and δ^18^O in soil water were categorized into two groups: one for δ^18^O < −10‰ and the one for otherwise, with the data in each group following its own linear relationship. The data in the above-part of the graph are for soil samples taken mainly from the 0–60 cm of soil, which can be fitted to a linear relationship, while the data for the 0–5 cm of soil in the graph fall below the δD - δ^18^O line for the stem water due to δ^18^O enrichment by evaporation. We linearly regressed the data taken from the 10–40-cm soil layer and plotted the results in Figure 2; it is close to the regression curve for the stem water.

**Figure 7:**
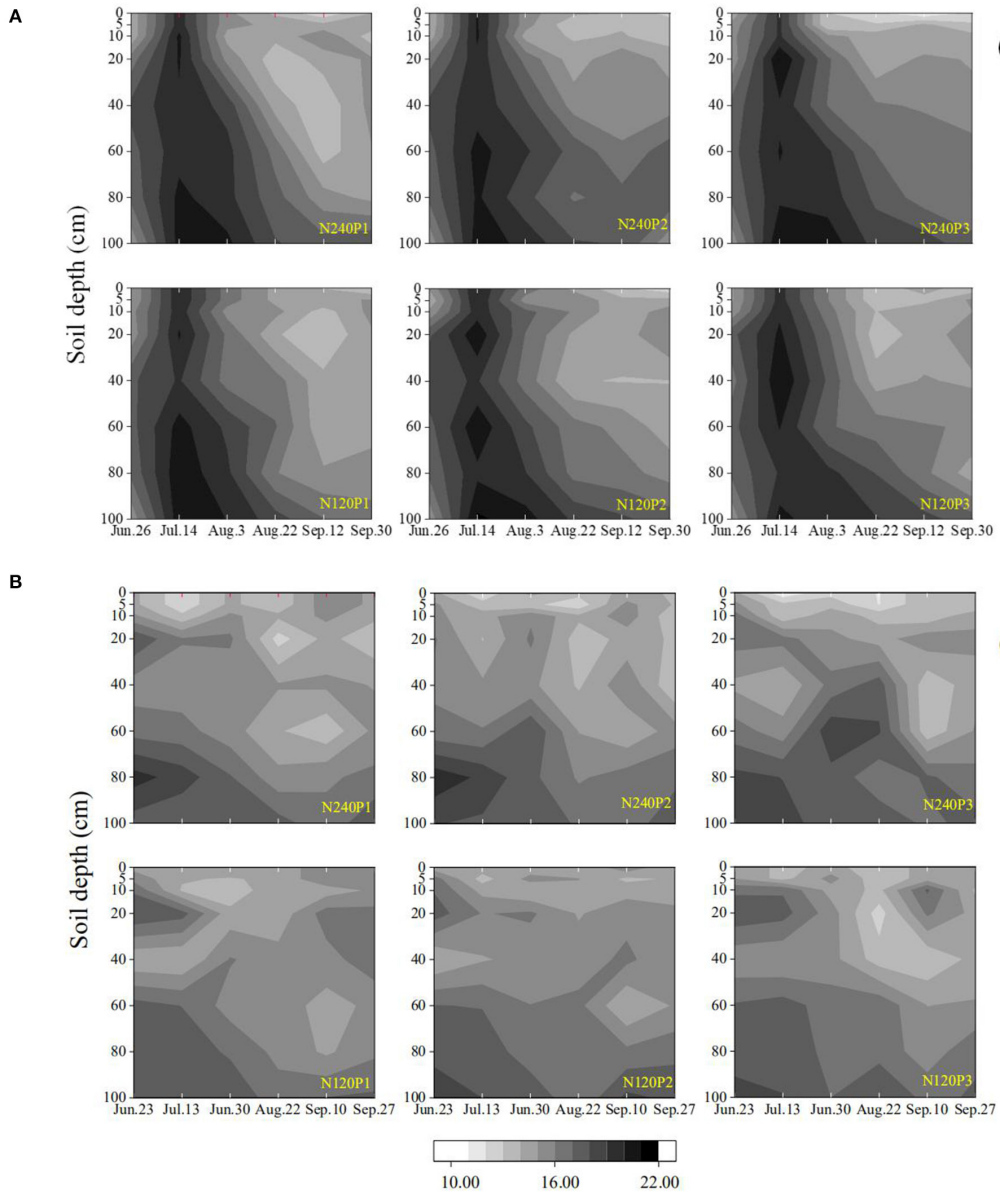
Spatiotemporal variations in soil moisture content under different treatments (see Figure 1A) in 2016 **(A)**, and 2017 **(B)**.

Soil evaporation enriched δ^18^O, thereby reducing the δD/δ^18^O ratio for soil samples taken from the regions proximal to the soil surface (Figure 2). In our results, soil water with low δ^18^O concentration was mainly for samples taken from the subsoil (60–100 cm). The likely mechanism is the attenuation of δ^18^O by molecular and hydrodynamic diffusion when it moves downward with the rainfall and irrigation water, which spreads the δ^18^O along the soil profile. Winter wheat–maize rotation is the main cropping system in the region, and before wheat harvest, the soil normally endures a prolonged drought which was likely to have enriched δ^18^O in soils proximal to the surface. In 2016, the 60 mm of irrigation after the seed drilling and the 123 mm of rainfall on July 9 (Figure 1B) combined to have leached the enriched δ^18^O below the depth of 100 cm as δ^18^O measured on July 14 was >-9‰ in most treatments, corroborated by the spatiotemporal changes in soil-water content (Figure 7). Although the enriched δ^18^O was likely to have moved upward since, driven by evaporation and root uptake, it remained in the soil deeper than 40 cm in most treatments (Supplementary Figures 1, 2), consistent with the spatiotemporal change in soil-water content (Figure 7) and the root water uptake (Figure 4). This is corroborated by the results in 2017 in which there was less rainfall and hence less water infiltration (Figure 1B). As a result, δ^18^O concentration in the subsoil is low and the δD/δ^18^O ratio is small compared to that in 2016. The δD - δ^18^O curve hence further deviates from the GMWL line (Figure 2B).

### Planting Pattern Effects

The planting pattern modulated the local environment for crops to grow both above-ground and below-ground (Dodd et al., 1998; Gani et al., 2002; Dass et al., 2015). Root-length density and root water uptake interactively affect each other (Coleman, 2007; Zhao et al., 2018), both varying with planting pattern and fertilization (Figure 4). Increasing planting density appeared to have enhanced water uptake by roots in the topsoil, especially under high N fertilization after the jointing stage (Figure 4). For example, in 2016, roots under combination of N_120_ and P1 took 25% of the transpired water from the soil around the depth of 60 cm, while under combination of N_240_ and P3, this soil layer supplied only 18% of the transpired water (Figure 4). Such variation in root water uptake with planting density was also found in other treatments in 2017, although the significance in the variation varies (Figure 4).

Roots regulate their water uptake from different soil layers as a response to change in soil water and other environmental factors (Figure 7). In P3, for example, the roots took 63% of the transpired water from the top 0–20 cm of soil on June 26, 2016. After the rainfall on July 19, the roots in the same soil layer contributed 67% to the transpired water despite the significant decrease in fraction of the root lengths in this soil layer (Figure 5). This increase in water uptake accompanied by a decrease in relative root length indicates that the roots in the top 0–20-cm soil layer were likely to have been water-stressed before the rainfall.

Compared to P1 and P2, P3 increased the root-length density in the top 0–10 cm of soil by 1.10-fold and 1.18-fold, respectively (Figure 5), similar to those found by others (Guan et al., 2007; Loades et al., 2010; Li et al., 2011). The consequence of the increased root density in the topsoil due to the increased planting density is that it increased the root water uptake and competition between roots for water in the topsoil (Tan et al., 2010; Li et al., 2011). Although the increased root water uptake depleted the topsoil water quickly, we did not find noticeable compensation by deep roots to increase their water uptake from the subsoil (Figure 4), indicating that the topsoil water remained sufficient for roots to take up and that the increased planting density did not enhance root penetration (Figure 5). The likely reason is that high planting density increased leaf area index thereby reducing water loss from evaporation (Hodge, 2004), as manifest from the soil-water change (Figure 7); this is consistent with the findings of others (Rossatto et al., 2013; Ma and Song, 2016).

Maize cultivars with shallow-root traits proliferate roots in the topsoil (Yi et al., 2009; Ma and Song, 2016), thereby increasing their water uptake from the topsoil (Schenk and Jackson, 2005). Zhao et al. (2018) suggested to express root water uptake as a function of dry-root weight rather than root-length distribution, claiming that the latter was inadequate to explain the spatiotemporal variation in root water uptake as often observed in the field (Ehleringer and Dawson, 1992). Physically, water acquisition by a root is driven by the difference between water potential at the root–soil surface and in the xylem network, and it depends on both root architecture and soil properties. Root traits that do not contain information on soil are hence insufficient to quantity root water uptake. Water needs to pass through the root surface prior to moving into the xylems, and the root-length density is hence one of the best proxies of root architecture to quantify root water uptake although root diameter and root age might also play an important part (Hodge, 2004). This is also corroborated by our results that the root water uptake increased asymptotically, rather than linearly, with the root-length density (Figure 6). The mechanisms underlying, such asymptotic increases, are manifold, including the variation in root diameter (hence the root–soil interfaces), which is not accounted for by the root-length density. The most likely reason, however, is the competition between roots in the topsoil for water, which reduces the uptake of individual roots (Wu et al., 2014). This is also consistent with previous studies on water acquisition of winter wheat where the root uptake rate is proportional to root-length density only when root-length density is <1 cm/cm^3^ (Gregory et al., 1978; Zhang et al., 2020).

### Effects of N Fertilization

Top-dressing N relieved root competition for N and hence enhanced root proliferation in the topsoil (Figure 5). On average, the roots in the top 20 cm of soil were longer under N_240_ than under N_120_ (Figure 5). The increased length of the shallow roots increased their water uptake from the topsoil (Figure 4). Depending on water availability in the topsoil, the roots regulate the ways they take up soil water. At the early growth stage, since the demand for water and nutrients was low and water and nutrients in the topsoil were sufficient, there was no noticeable difference in root water uptake between treatments (Figure 4). As crops grew and their demands for water and nutrients increased, the differences in root water uptake between treatments emerged (Figure 4).

As the planting density increases, the demand for water and nutrients increases. If one or two of them becomes limiting, the roots penetrate to access water and nutrients in the subsoil (Rogers and Benfey, 2015). Our results showed that when the planting density was the same, the roots took more water from the subsoil under low N fertilization (Figure 4). Denser planting density associates with high root-length density and needs more water, especially in the topsoil. The fact that the root water uptake from the subsoil is affected by N more than by the planting density implies that N was more likely the limiting factor in our experiment (Figure 4). For example, roots in the combination of P3 and N_240_ took approximately 35% of the transpired water from the top 0–15 cm of soil (Figure 4), while the roots in the same soil layer took only 30% of the transpired after halving the N application (Figure 4). This is corroborated by the recent finding of Ma and Song (2016) where the roots tended to exploit water and nutrients from the deep soil when fertilizer was in deficiency. The results of Ma and Song (2016) showed that the root water uptake increased linearly with root-length density, indicating the absence of water stress and root competition. This differs from our experiment where there was a slight water stress in the topsoil, and the root water uptake hence increases asymptotically with the root-length density (Figure 6).

Developing sustainable agriculture in arid and semi-arid regions, such as northern China (Guan et al., 2015), requires improving the use efficiency of soil water and rainfalls (Fang et al., 2010). While various efforts have been made, the efficacy of manipulating planting density and fertilization to help achieve this goal has been overlooked. On average, maize planted in high density in the zig-zag pattern took more water from the topsoil than from the subsoil due to the proliferation of shallow roots, especially when combined with high N application (Figure 5). It thus improves rainfall use efficiency as it preferentially uses the topsoil water, consistent with other studies (Hatfield et al., 2001; Zhang et al., 2005).

## Conclusion

Spatiotemporal change in root water uptake of summer maize was studied in a 2-year field experiment comprising three planting patterns and two N fertilizations. Water uptake by roots at different soil layers was calculated using stable isotopes δD and δ^18^O measured concurrently from soil water and crop stem. The results showed that the crop took most of the required water from the 0–60 cm of soil, but the uptake pattern varied with treatments. Regardless of the planting patterns, reducing N fertilization boosted root penetration to access nutrients in the subsoil and consequently increased the root water uptake from the subsoil. Increasing planting density and uniformity enhanced proliferation of shallow roots and their water uptake from the topsoil due to the increased leaf index area which reduced water loss from evaporation, especially in the early growth stage. In soil profile, because of the competition of shallow roots for water, root water uptake increased asymptotically rather than linearly with root-length density. In summary, high planting density combined with high N fertilization improves the preferential use of the topsoil water which is prone to evaporation and is hence more water-use-efficient for rain-fed maize production in semi-arid regions.

## Data Availability Statement

The raw data supporting the conclusions of this article will be made available by the authors, without undue reservation.

## Author Contributions

YG: funding acquisition and writing—original draft preparation. JC: investigation. GW: visualization. ZL: data curation and software. YZ and WS: investigation and data curation. XZ: methodology and writing—reviewing and editing. All authors contributed to the article and approved the submitted version.

## Funding

This research was supported by the China Agriculture Research System (CARS-02) and the National Natural Science Foundation of China (51879267). The work at Rothamsted Research was supported by the United Kingdom Biotechnology and Biological Science Research Council (BBSRC)-funded Soil to Nutrition strategic program (BBS/E/C/000I0310).

## Conflict of Interest

The authors declare that the research was conducted in the absence of any commercial or financial relationships that could be construed as a potential conflict of interest.

## Publisher's Note

All claims expressed in this article are solely those of the authors and do not necessarily represent those of their affiliated organizations, or those of the publisher, the editors and the reviewers. Any product that may be evaluated in this article, or claim that may be made by its manufacturer, is not guaranteed or endorsed by the publisher.
