# Supplementary figures and images for: Different Responses in Root Water Uptake of Summer Maize to Planting Density and Nitrogen Fertilization
Source: Front Plant Sci. 2022 Jun 24;13:918043. doi: 10.3389/fpls.2022.918043 (PMC9263914; doi:10.3389/fpls.2022.918043)

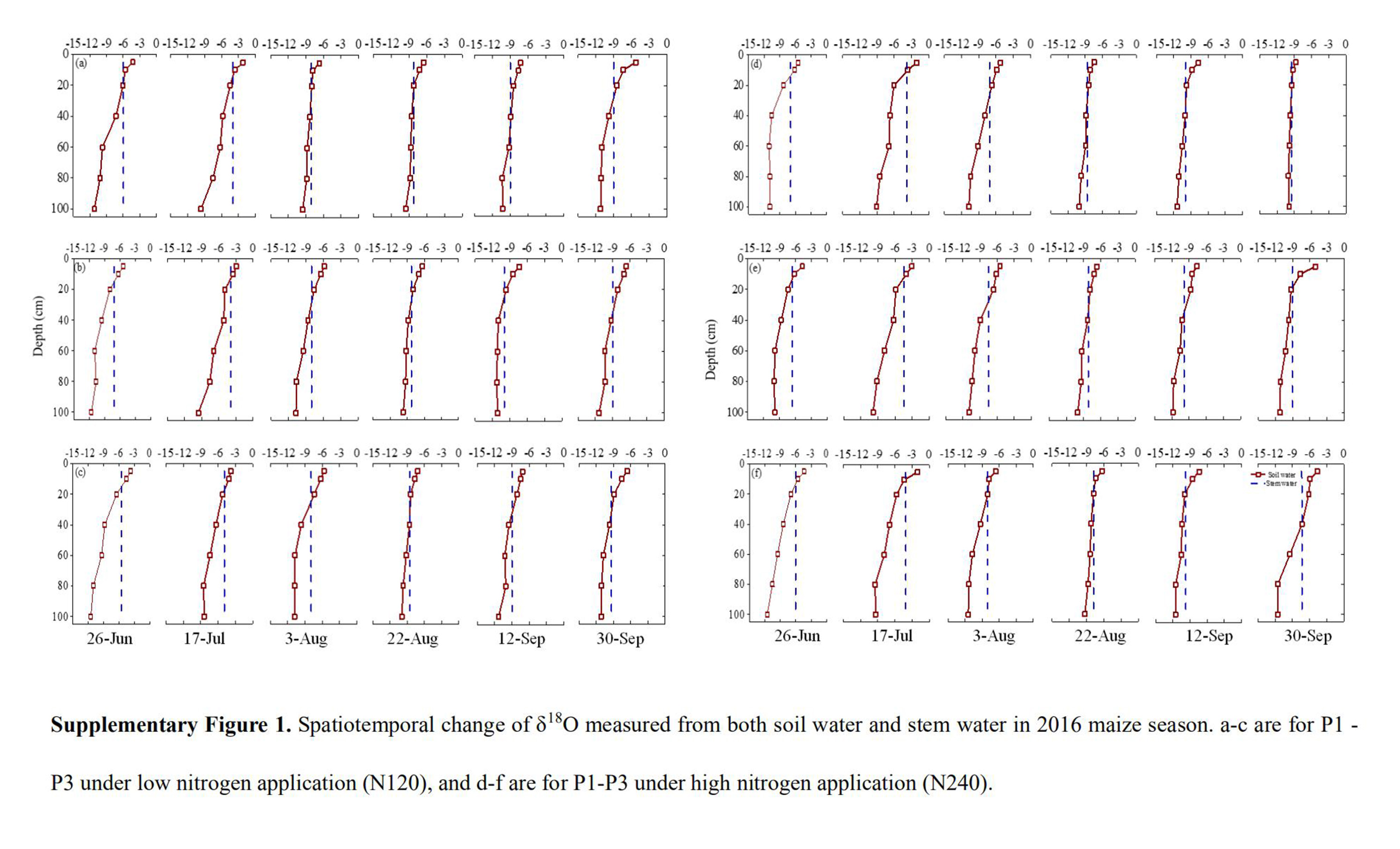

Supplement: Supplementary file 1 [file Image_1.jpeg]

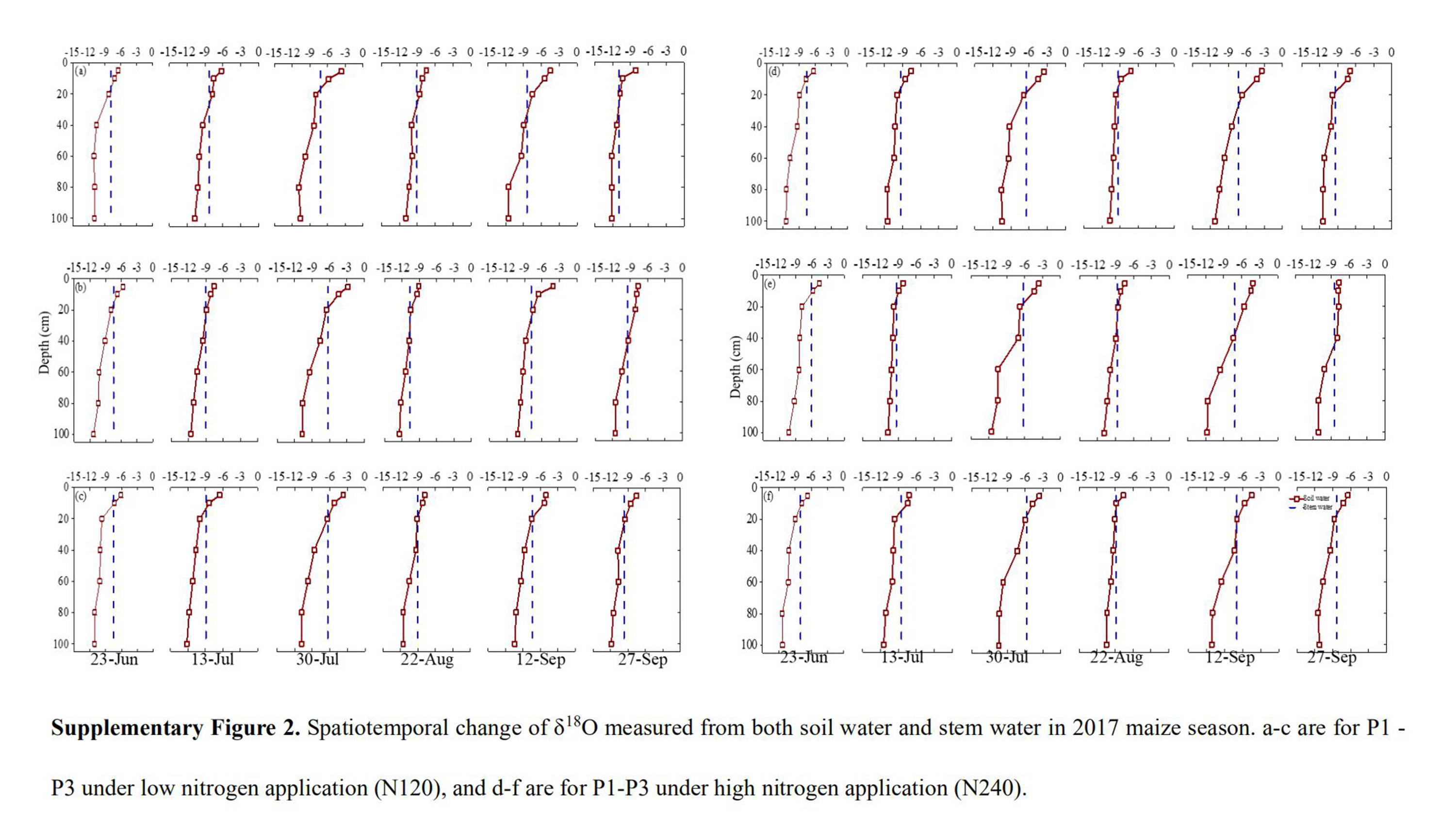

Supplement: Supplementary file 2 [file Image_2.jpeg]
